# Determinants of clean birthing practices in low- and middle-income countries: a scoping review

**DOI:** 10.1186/s12889-020-8431-4

**Published:** 2020-05-01

**Authors:** Joanna Esteves Mills, Erin Flynn, Oliver Cumming, Robert Dreibelbis

**Affiliations:** 1 Disease Control Department, London School of Hygiene and Tropical Medicine, Keppel Street, London, WC1E 7HT UK; 2 Infection & Immunity, South Australian Health and Medical Research Institute, North Terrace, Adelaide, 5000 Australia

**Keywords:** Maternal, Neonatal, Health, Clean, WASH, Hygiene, Labour, Birth, Delivery, Peri-natal, Determinants

## Abstract

**Background:**

Infection is a leading cause of maternal and newborn mortality in low- and middle-income countries (LMIC). Clean birthing practices are fundamental to infection prevention efforts, but these are inadequate in LMIC. This scoping study reviews the literature on studies that describe determinants of clean birthing practices of healthcare workers or mothers during the perinatal period in LMIC.

**Methods:**

We reviewed literature published between January 2000 and February 2018 providing information on behaviour change interventions, behaviours or behavioural determinants during the perinatal period in LMIC. Following a multi-stage screening process, we extracted key data manually from studies. We mapped identified determinants according to the COM-B behavioural framework, which posits that behaviour is shaped by three categories of determinants – capability, opportunity and motivation.

**Results:**

Seventy-eight studies were included in the review: 47 observational studies and 31 studies evaluating an intervention. 51% had a household or community focus, 28% had a healthcare facility focus and 21% focused on both. We identified 31 determinants of clean birthing practices. Determinants related to clean birthing practices as a generalised set of behaviours featured in 50 studies; determinants related specifically to one or more of six predefined behaviours – commonly referred to as “the six cleans” – featured in 31 studies. Determinants of hand hygiene (*n* = 13) and clean cord care (*n* = 11) were most commonly reported. Reported determinants across all studies clustered around psychological capability (knowledge) and physical opportunity (access to resources). However, greater heterogeneity in reported behavioural determinants was found across studies investigating specific clean birthing practices compared to those studying clean birthing as a generalised set of behaviours.

**Conclusions:**

Efforts to combine clean birthing practices into a single suite of behaviours – such as the “six cleans”– may simplify policy and advocacy efforts. However, each clean practice has a unique set of determinants and understanding what drives or hinders the adoption of these individual practices is critical to designing more effective interventions to improve hygiene behaviours and neonatal and maternal health outcomes in LMIC. Current understanding in this regard remains limited. More theory-grounded formative research is required to understand motivators and social influences across different contexts.

## Background

Childbirth, and the days immediately after, are a particularly vulnerable time for mothers and their newborns. Globally, an estimated three million babies die each year in the first month of life [1] Infections, including sepsis, meningitis, pneumonia, diarrhoea and tetanus, account for approximately one-quarter of all deaths during the neonatal period (the first 28 days of a child’s life); 30–40% of infections resulting in neonatal sepsis deaths are estimated to be transmitted during childbirth [2].

There has been significant effort in many low- and middle-income countries (LMIC) in the last two decades to increase the proportion of births taking place in health care facilities (HCF) [3]. This is to ensure that women and their newborns are attended by skilled personnel and have access to life saving care if complications arise. Recent analysis of birthing trends using data from multiple household surveys shows that globally the proportion of births taking place in healthcare facilities has increased from 52% in 2000 to 76% in 2018 [3]. According to a recent analysis by Montagu and colleagues, this increase has been observed across all geographic regions, urban and rural populations, and for public and private facilities [4]. However, newborns and mothers in LMICs remain at greater risk of healthcare-associated infections compared to their counterparts in high income countries [5, 6]. Hygiene practices during labour, delivery and post-natal care – referred to here as “clean birthing” practices – are critical to the reduction of maternal and neonatal infections [7–9]. There are a range of specific behaviours that fall within this definition of clean birthing practices, including: clean hands of the birth attendant, clean delivery surface, clean perineum, cutting of the umbilical cord using a clean instrument; clean cord tie; and clean cord care [10, 11]. These practices are commonly referred to as the “six cleans” [7].

Despite global guidelines and promotion, several studies have identified inadequate adoption of clean birthing practices at both the institutional [12–14] and community-level in LMIC [15, 16]. Systematic investigation into clean birthing practices in LMIC has to-date been concentrated on umbilical cord care [17, 18] and hygiene among traditional birth attendants [19]. More recently, there has been increased attention to the availability and quality of water, sanitation, and hygiene (WASH) infrastructure in health care facilities [20–22]. These are fundamental in facilitating hygienic birth and postnatal care, and are essential for the delivery of most infection prevention control procedures and quality of care more generally [23]. However, even in the presence of improved WASH infrastructure, babies and mothers remain at risk of infection when clean birthing practices are sub-optimal [24].

Guidelines for the development of interventions to improve hygiene in healthcare facilities recommend that interventions should be informed by the requirements of the target population, current practices and preferences, and barriers and drivers of desired behaviours [23]. The objective of this scoping study is to synthesize available literature on the individual, social, and physical environmental determinants of clean birthing practices during labour, delivery and the period immediately after (the peri-natal period) and how these determinants have been addressed by interventions.

## Methods

We adapted Arksey and O’Malley’s scoping study methodology [25] to review both qualitative and quantitative studies describing individual, social, and environmental drivers of hygiene behaviours of healthcare workers or mothers during the peri-natal period, including both home and facility deliveries.

Two databases (PubMed and Web of Science) were used in March 2017 to identify relevant studies published in English between 2000 and February 2017. Search strings included key words and subject headings associated with the following terms: behaviours, attitudes, knowledge, practices AND hygiene, handwashing, clean, cord cutting, cord care, delivery surface, perineum AND newborn care, childbirth, obstetric care. Studies published prior to 2000 were excluded based on resources available for screening and reviewing citations. Searches were repeated in February 2018 to identify new manuscripts published since the initial search.

We followed a multi-stage screening process, applied first to title, then abstract, then full text. Studies were included if they provided qualitative or quantitative information on behaviour change interventions, behaviours or behavioural drivers related to hygienic practices during labour, delivery, and post/neonatal periods. The exclusion criteria were: studies outside the labour, delivery and immediate post-natal period, studies not conducted in LMIC, and studies reporting on interventions that did not include a behaviour change component or insights into clean birthing practices. Systematic reviews, editorials, and opinion articles were also excluded. Studies that focused on outbreak investigations of specific pathogens and/or specific environments – such as neonatal intensive care units were considered beyond the scope of the current study and excluded.

For the charting phase of our study, key data were extracted manually from all studies in the form of a purposefully designed Microsoft Excel database (Microsoft, Redmond WA, USA). Data extracted included information on study design and objectives, target population, intervention description, outcome measures, analysis and results.

This qualitative meta-analysis followed a multi-stage process to identify and classify published information on determinants of clean birthing practices. First, we reviewed published manuscripts and extracted information on clean birthing practices and associated determinants. We defined determinants broadly as any single or group of factors that influenced the likelihood of a defined behavioural outcome. Predefined behaviours included: hand washing, clean delivery surface, clean perineum, cord cutting, cord tying and cord care. For every manuscript reviewed we attributed each identified determinant to one of the specific clean birthing behaviours of interest. Within each study, several determinants could be associated with a single, specific behavioural outcome and several individual behaviours could be associated with the same determinant. Data on all available pairwise determinant/behaviour relationships were included in the analysis. Several manuscripts did not provide data on individual behaviours, but rather described clean birthing practices as a generalised set of behaviours (for example, “the six cleans” or “clean birthing practices”). For these studies, we assigned identified determinants to “general clean birthing practices”.

Identified determinants were then classified into six categories based on the proximal determinants of behaviour presented in the COM-B Framework. This framework provides a structured system for categorizing a wide-range of potential determinants that is applicable across behaviours and study designs [26] (Table 1). Broadly speaking, COM-B posits that behaviour is shaped by three categories of determinants – capability, opportunity, and motivation. Capability is defined as “the individual’s psychological and physical capacity to engage in the activity concerned” [26]. Opportunity is defined as “all the factors that lie outside the individual that make the behaviour possible or prompt it” [26] and consists of both social opportunity and physical opportunity. Motivation is defined as, “all those brain processes that energize and direct behaviour, not just goals and conscious decision-making” [26] and includes both reflective (conscious intentions or plans) and automatic motivation (emotions and impulses).

**Table 1:** COM-B Model

**Capability**	The individual’s psychological and physical capacity to engage in the activity concerned.	*Psychological*	The capacity to engage in the necessary thought processes - comprehension, reasoning et al.
		*Physical*	Physical capacity to engage in the activity concerned. Includes having the necessary skills.
**Motivation**	All brain processes that energise and direct behaviour, not just goals and conscious decision-making.	*Reflective*	Reflective processes that involve evaluations and plans.
		*Automatic*	Automatic processes that involve emotions, impulses that arise from associative learning, and/or innate dispositions.
**Opportunity**	All the factors that lie outside the individual that make the behaviour possible or prompt it.	*Social*	Social opportunity afforded by the cultural milieu that dictates the way that we think about things (e.g. the words and concepts that make up our language).
		*Physical*	Physical opportunity afforded by the environment.

In a final step, determinants identified in intervention studies were classified according to whether they were explicitly identified or implicit from the intervention design or findings. By ‘explicit’ we mean that the authors either articulated a full theory of change for their target behaviour(s) and that the described intervention matched with that, or that authors explicitly identified the determinants targeted by their intervention. For the remaining studies, we identified determinants implicit in the scope and nature of the intervention described. For example, a study assessing the effectiveness of a predominantly directive educational hand-hygiene intervention may not explicitly identify drivers or barriers, but the importance given to knowledge as a driver for the target behaviour is implicit in the intervention design.

The coding was conducted primarily by one researcher, with spot checking carried out by a second researcher. Following the abstract review stage, the second researcher selected 10% of the remaining papers for full article review using a random number generator. Using the same extraction tools, she independently mapped the data and classified the determinants for these papers. A third researcher cross-checked the data extracted and the decisions made on classification for this sub-section of papers. This was particularly important for the inherently subjective classification of determinants according to the COM-B categories. For those papers where there was disagreement between the first and second researcher, the third reviewer charted the paper independently. There were no examples of papers where there were three distinct categorisations so all discrepancies were resolved in this manner.

## Results

We retrieved 4932 articles, of which 1225 were duplicates. After screening records by title and abstract, 165 studies were retained. Papers that focused on Chlorhexidine (CHX) use (*n* = 41) were excluded at this stage on the basis that a large number of existing systematic reviews and meta-analyses have been published specifically on CHX [18, 27–29]. At full text review, a further 85 manuscripts were excluded, as they did not meet the inclusion criteria and four full text versions could not be sourced.

Searches repeated in February 2018, to capture new publications, retrieved another 480 papers, resulting in 26 additional manuscripts included in synthesis. An additional four papers were identified as a result of mining references of relevant manuscripts.

**Fig. 1:**
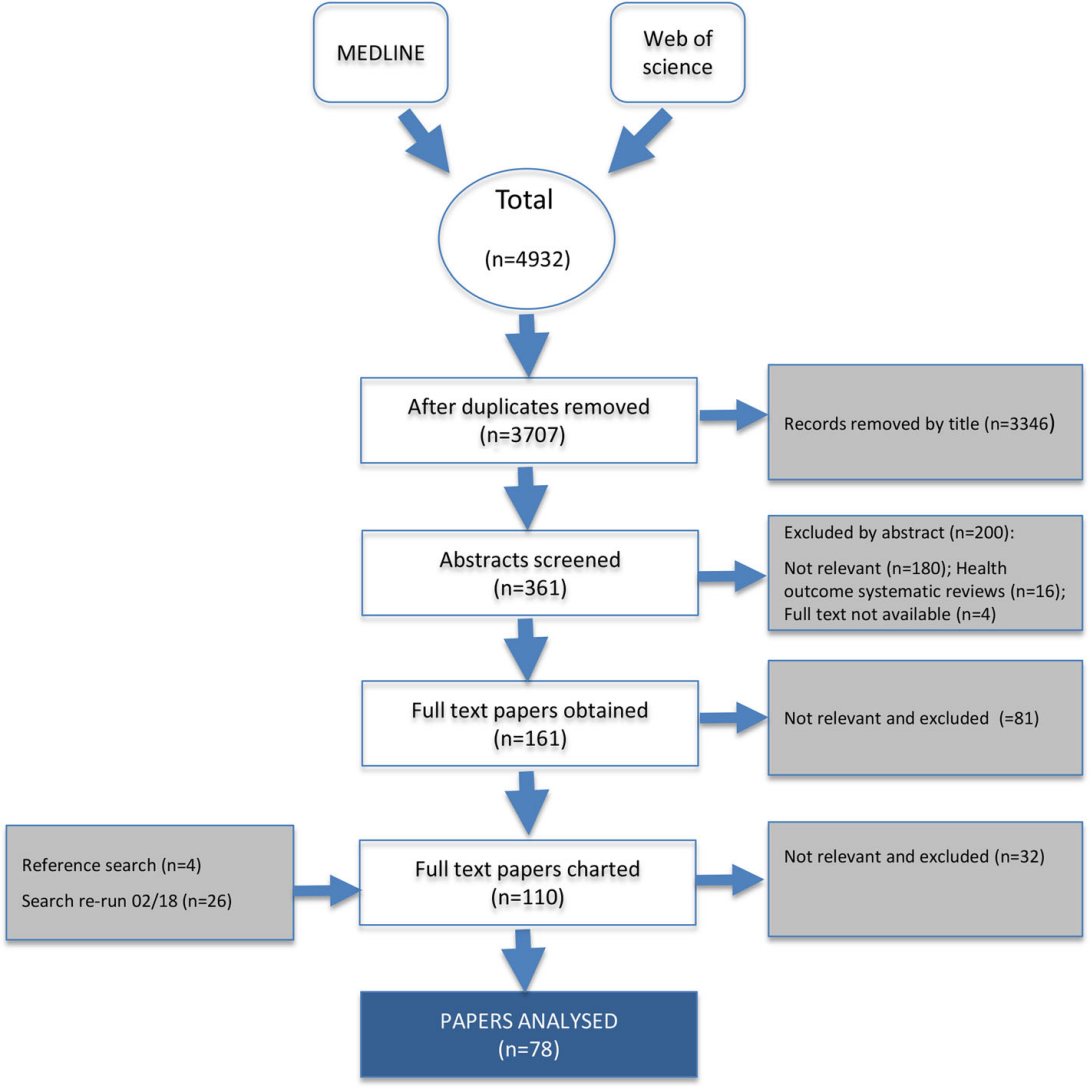
Review results

Data were extracted from a total of 110 manuscripts. During synthesis, an additional 32 were excluded based on relevance. A total of 78 papers were included for analysis. See Fig. 1 for details.

### Study focus and design

Among included studies, 60% (47/78) were observational studies [30] while 40% (31/78) described an evaluation of intervention activities (referred to from now on as intervention studies). Among the 31 studies that assessed an intervention, approximately half (16/31) included a control or comparison group. Among all studies, 55% were conducted in Asia (43/78), 40% in Africa (31/78) and 4% (3/78) in Central America and the Caribbean. Half of the studies (51%; 40/78) had a household or community focus compared to 28% (22/78) focusing at the health care facility level and 21% of studies (16/78) focused across both environments. The 31 intervention studies were distributed evenly across health facilities (12/31), domestic environments (11/31), or both (8/31).

### Behavioural determinants

Across all studies, we identified 31 determinants of clean birthing practices, either explicitly articulated in the published manuscript or extrapolated based on intervention/study design. Determinants identified in the literature are mapped against their associated COM-B category in Additional file 1. We identified behavioural determinants related to general clean birthing practices in 64% of reviewed studies (50/78) and determinants related to one or more of five specific clean behaviours in 50% (39/78): hand hygiene (of the attendant or the caregiver), clean delivery surface, clean cord-cutting instrument, clean cord tie, clean cord care. Determinants were not reported regarding cleaning of the perineum. Of those studies where it was possible to identify determinants for individual clean practices, hand hygiene (13/39) and clean cord care (11/39) were most commonly reported (Table 2).

**Table 2:** Studies with information on behavioural determinants for specified clean birthing practices published January 2000 – February 2018

Hygienic birth practices	No. of studies
General clean birthing practices	50
Hand hygiene	13
*Attendant*	*9*
*Caregiver*	*4*
Clean delivery surface	5
Clean cord-cutting instrument	5
Clean cord tie	5
Clean cord care	11

#### Determinants identified in observational studies

The frequency with which determinants were identified across the 47 observational studies reviewed is recorded in Table 3. A more detailed version of this table that includes study references is in Additional file 2. In sum, 49% of the observational studies reviewed (23/47) identify determinants associated with general clean birthing practices. A total of 15 determinants for this set of generalised clean birthing practices were identified across these studies. Six specific determinants related to capability were identified, with knowledge (13/23) and skills (8/23) receiving by far the most attention across studies. Five studies identified inadequate training of birth attendants, reported to result in limited knowledge and skills related to clean birthing practices [31–35]. Two studies (one focusing on home births and the other on facility based births) present positive associations between the adoption of clean birthing practices and mothers with higher educational levels [36], current employment or having 2–3 children [37]. While this is not explicitly stated, it is possible that these determinants are being used as proxies for maternal knowledge or socio-economic status of the mother, all inextricably bound. One study described how the availability of national newborn care guidelines were an important predictor of good newborn care practices by health care workers, including appropriate cord tying and cord care [32].

**Table 3:** Frequency of mention of determinants among observational studies (*n* = 47)

	Gen.	Hand hygiene		Surface	Blade	Tie	C. Care
	*n = 23*	*Attendant n = 7*	*Caregiver n = 3.*	*n = 5*	*n = 4*	*n = 5*	*n = 10*
**Capability**							
Psychological							
Education	1						1
Guidelines	1	1					
Knowledge	13	4	3		3	4	4
Occupation	1						
Parity	1						
Physical							
Skills	8				1	1	
**Motivation**							
Reflective							
Product acceptability							1
Futility	1						
Practical considerations				4			
Willingness to pay							1
Product cost	1						
Automatic							
Automaticity			1				
Disgust			1				
Nurture			1			1	4
Sense of ownership							1
Sense of pride	1						
**Opportunity**							
Social							
Soc. hierarchies/ community influencers	2	1	1	1			4
Traditional/cultural beliefs	5			4	2	1	5
Physical							
Adequate materials/supplies	7	5				1	
Convenience	1	2					
Infrastructure	4	3					
Time	1	1	1	1			

A total of six specific determinants related to opportunity were identified in 14 observational studies (14/47). Physical opportunity, more specifically access to adequate materials such as soap or clean delivery kits (7/14) and water, sanitation and/or hygiene infrastructure (4/14), received the greatest attention. One study identified time as a barrier to improved practices [38]. The importance of traditional/cultural beliefs – a component of social opportunity – was described in five studies. One study in south-eastern Nigeria reports that knowledge of and familiarity with cultural practices, as well as the ability to offer comprehensive care, made traditional birth attendants (TBAs) a popular option for care during delivery in their local community [39]. In this study TBAs had good knowledge of the importance of personal hygiene, including hand washing, during the antenatal period. Another study in Karamoja, Uganda, reports that birth preparedness is restricted by a local tradition that forbids women to buy delivery supplies before the baby is delivered [40].

Determinants associated with motivation receive relatively little mention in the 23 observational studies reporting on general clean birthing practices, with only four studies providing information relative to motivational determinants. In Cambodia, skilled birth attendants viewed attempts at cleanliness as pointless, due to factors such as the unclean clothing of labouring women [38]. In rural Tanzania, women reported the costs of materials for clean birthing practices in a home delivery – razor blade, thread and gloves – as a barrier to adoption of these practices [41].

57% of observational studies reviewed (27/47) identify determinants for a specific clean practice, and, of these, the majority focus on hand hygiene (10/27) or clean cord care (10/27), followed by clean cutting or tying of the umbilical cord (7/27) and clean birthing surface (5/27). Among the 10 hand hygiene studies, seven focus on hand hygiene of the birth attendant and three focus on hand hygiene of other caregivers. Studies on birth attendant hand hygiene focus mostly on health care facility staff (5/7) with only two studies providing information specific to hand hygiene among birth attendants in domestic environments. For attendant hand hygiene, identified determinants most commonly relate to physical opportunity, with six studies providing information on four specific physical opportunity determinants, including supply of materials (5/7), convenience (2/7), infrastructure (3/7) and time (1/7). Psychological capability – namely knowledge (4/7) and guidelines (1/7) – was the second most common category of determinant for attendant hand hygiene. Knowledge was the most commonly cited determinant of caregiver hand hygiene (3/3). There were only a limited number of studies that addressed caregiver opportunity or motivation for improved hand hygiene. One study in Indonesia [15] described the motivational drivers of handwashing behaviour among new mothers, namely disgust, for example after changing a soiled nappy, as well as nurture or the desire to care for their newborn. In a study of new mothers in India, 15% of their interviewees said they had no time for proper handwashing [42].

Only five observational studies included information related to the determinants of clean birthing surfaces (5/47). A total of four determinants were identified across these studies, most commonly related to social opportunity. Specifically, these studies identified the common belief that childbirth – and by extension the mother and neonate – is polluted and/or impure and that it is not worth using clean materials to protect them. Reflective motivation was also a determinant of clean birthing surfaces, specifically considerations about which surfaces are easier to clean and/or dispose of following childbirth. One study in a domestic setting in Bangladesh reports that women delivered on the floor, on a jute bag, or on straw as this made cleaning and disposing of impure blood and placenta easier [43]. Khadduri et al. describe how the practice of placing a plastic sheet under the mother in Pakistan was rarely done with clean plastic and was often intended to protect the surface not the mother or child [44].

Seven observational studies (7/47) identified a total of four determinants for clean cord cutting and/or tying. Determinants of these practices were often presented combined. Psychological capability (in particular insufficient knowledge) is the most commonly reported barrier (5/7). This relates to both mothers, who are responsible for preparing thread and blade prior to delivery, and TBAs, who tie the cord and cut it. One study specified that while the importance of handwashing and germs was widely understood with regards to cord care, the importance of a clean blade was not common knowledge [44]. Another study found a significant association between the use of antenatal care (ANC) and the use of a clean blade and tie, suggesting that exposure to ANC was associated with knowledge of the optimal behaviours [45]. Social opportunity (in particular cultural or traditional beliefs) was described as a barrier to clean cord cutting/tying in three studies. For example, one study reports that TBAs tie the cord in the belief that it prevents air from entering the baby [46].

Ten observational studies (10/47) found a total of eight determinants of clean cord care. The determinants most commonly referred to were traditional or cultural beliefs (5/10) and community influencers (4/10), both of which fall under the social opportunity category of determinant. For example, the cultural belief that the cord is harmful to the newborn as a channel for witchcraft or evil spirits is reported in two studies in Tanzania and Haiti as driving the mothers to use sometimes harmful substances on the cord to speed up the drying process [41, 47]. Nurture is often the implied deeper determinant of such culturally informed practices, as it is believed that the longer the cord is attached the more vulnerable the child is to either health or spiritual risks. This is perhaps clearest in a paper reporting that women apply different cord care practices according to the sex of their new offspring. If it is a boy, mothers are more likely to practice cord-care-related behaviours that, while posing a health risk, respond to their heightened desire to nurture [48]. The influence of senior community figures, including grandmothers and traditional healers and birth attendants, is highlighted in four studies [46–49]. One observational study in Nigeria notes how this influence, especially influence by attending nurses, mother or mother-in-law, can supersede a woman’s own knowledge of the risks or benefits associated with a given practice [48].

#### Determinants identified in intervention studies

Only 35% (11/31) of the behaviour change intervention studies identified in this review are explicit about which behavioural determinant(s) their intervention targeted. For the remaining studies (20/31), information on targeted behavioural determinants was inferred based on intervention descriptions.

Details of the 20 interventions where the target determinants are not explicitly identified are recorded in Additional file 3, including intervention aim, activities, expected outputs, and implied determinants. Two studies provided information on the determinants of use of maternal health services in Kenya and India. In these studies, clean birthing practices are measured as outcomes associated with accessing maternal health services; however, the mechanisms through which this occurs are not articulated [50, 51].

Among the remaining studies, the majority (15/20) involve educational messaging to the mother or TBA about maternal and newborn clean care behaviours. Of interventions that provided education, 40% (6/15) also distributed essential materials, most commonly a clean delivery kit, to enable desired behaviours. This intervention design implies that the barriers to adoption of clean birthing practices are insufficient knowledge and inadequate access to necessary materials. One intervention distributed materials (including soap and clean delivery kits) at ANC visits, and these products were found to successfully incentivise mothers to obtain ANC, suggesting the importance of motivational determinants [50]. Four studies (4/15) described interventions that included a community engagement component in addition to the standard educational messaging to mother or TBA. These included efforts to engage local leaders and/or community elders through community health committees or existing community structures [51–54]. The inference here is that the interventions were specifically targeting determinants related to social opportunity.

Five studies (5/20) described multi-modal interventions within a health care facility, either for general quality improvement [55–57] or specifically to improve adherence to the WHO Safe Childbirth Checklist [58, 59]. The interventions typically included training of health care staff, engagement of senior facility and district personnel, continual monitoring, feedback and action cycles, and mentoring, coaching and supervision of staff. These components suggest the significance of knowledge and skills, job motivation and the fear of repercussions as main drivers of behaviour.

Of the eleven studies that are explicit about which determinant(s) the interventions targeted (11/31), seven report on target determinants of clean birthing practices in general and only four identify target determinants specific to a particular practice. Details of the interventions explicitly targeting identified determinants for clean practices in general are recorded in Additional file 4. There are three studies (3/7) of community-based interventions [60–62] and three studies (3/7) of healthcare facility-based interventions [63–65]. One study (1/7) evaluated a community and facility-based intervention [66]. The most commonly targeted determinant was knowledge of the mother or birth attendant, with four interventions (4/7) aiming to improve knowledge through increased use of maternal and newborn services [66], home-based maternal counselling by community volunteers [60], improved quality of facility-based maternal counselling through use of visual job-aids [65], or facility-based training of care providers on essential newborn care practices [64]. Two focused on participatory approaches, specifically the active involvement of women, families, and community members in effecting behaviour change [61, 62]. Two interventions engaged powerful community members in influencing maternal or attendant behaviours. Job motivation emerges as a minor theme, featuring in one healthcare facility-based study in India that included peer coaching and performance monitoring [63]. Another minor theme, featuring in one community-based study, was utilizing a woman’s life-stage – namely pregnancy – as a teachable moment [60].

Details of the four interventions (4/31) explicitly targeting identified determinants for specific clean birthing practices are recorded in Additional file 5. The four interventions targeted clean hands of the attendant (2/4) [67, 68] and of the carer (1/4) [16], clean blade (1/4) [67], and cord care (1/4) [69]. Knowledge is identified as a determining factor for attendant handwashing with soap in a healthcare facility setting in Nicaragua [68] and for using a clean blade to cut the umbilical cord in a community setting in India [67]. In Nicaragua, the absence of alcohol gel was also highlighted as a barrier, leading to an intervention based on guideline development, training of medical staff and revision of the basic medical supply list. In India, the importance of social norms and collective behaviours, the impact of community influencers and decision-makers was also raised, leading to a community-based intervention delivered by community health workers with a multi-level strategy for engagement of individuals with key roles as influencers, decision makers, supporters, practitioners of newborn care and normative behaviour within community. This community approach also targets a further determinant identified in this study; the role of cultural beliefs in shaping hand hygiene behaviour among attendants. The delivery process and the newborn are reportedly unclean and ‘polluting’ so birth attendants usually do not see the value in handwashing. Cultural beliefs also emerge as an important barrier to clean cord care practices in a Maasai community in East Africa, which reports that the application of cow dung to the cord is culturally symbolic, underlining the close connection between the Maasai way of life and the tending of cattle [69]. The importance of participatory approaches in effecting caregiver hand hygiene behaviour change, as well as the significance of the pregnancy period as a teachable moment, were identified in one facility-based study [16], which responded with an interactive educational intervention to promote handwashing, using behaviour change communicators trained on motivational interviewing to encourage active engagement from participants and their families.

#### Behavioural determinants summary

Table 4 illustrates the frequency with which COM-B categories feature across the entire body of literature included in this review. Generally, determinants cluster around psychological capability (most commonly knowledge) and physical opportunity (most commonly access to resources). However, the picture is more nuanced when we disaggregate according to specific behaviours of interest. For example, among 13 studies that focus on determinants of hand hygiene the most investigated determinants are knowledge (*n* = 8, 62%), materials/supplies (*n* = 6, 46%) and infrastructure (*n* = 3, 23%), which is in line with studies that identify determinants for clean birthing practices in general. However, when the analysis is restricted to studies that identify determinants for clean cord care (*n* = 11), the importance of social hierarchies/community influencers (*n* = 4) and traditional or cultural beliefs (n = 6) gain markedly in prominence relative to knowledge.

**Table 4:** Determinants using COM-B classification according to frequency with which they feature in the literature reviewed

	General	Hand hygiene		Clean delivery surface	Clean cord cutting instrument	Clean cord tie	Clean cord care	Total
		***Attendant***	***Caregiver***					
	***n = 50***	***n = 9***	***n = 4***	***n = 5***	***n = 5***	***n = 5***	***n = 11***	
**Capability**								
Psychological								
Confidence	1	0	0	0	0	0	0	1
Educational level	1	0	0	0	0	0	1	2
Experience	0	0	0	0	0	0	0	0
Guidelines	1	1	0	0	0	0	0	2
Knowledge	36	5	3	0	4	4	4	56
Occupation	1	0	0	0	0	0	0	1
Parity - no. children	1	0	0	0	0	0	0	1
Physical								
Skills	16	0	0	0	2	1	0	19
**Motivation**								
Reflective								
Product acceptability	0	0	0	0	0	0	1	1
Futility	1	0	0	0	0	0	0	1
Impunity	4	0	0	0	0	0	0	4
Fear of repercussions	0	0	0	0	0	0	0	0
Practical considerations	0	0	0	4	0	0	0	4
Willingness to pay	0	0	0	0	0	0	1	1
Product cost	1	0	0	0	0	0	0	1
Automatic								
Automaticity	0	0	1	0	0	0	0	1
Disgust	0	0	1	0	0	0	0	1
Job motivation	3	0	0	0	0	0	0	3
Nurture	0	0	1	0	0	1	4	6
Ownership/participatory approaches	5	0	1	0	0	0	1	7
Sense of pride	1	0	0	0	0	0	0	1
Teachable moment	2	0	1	0	0	0	0	3
**Opportunity**								
Social								
Collective behaviours/social norms	0	0	0	0	0	0	0	0
Social heirarchies/community influencers	7	2	1	1	1	0	4	16
Traditional/cultural beliefs	5	1	0	4	2	1	6	19
Trust in attendant	1	0	0	0	0	0	0	1
Physical								
Adequate materials/supplies	15	6	0	0	0	1	0	22
Convenience of the activity	1	2	0	0	0	0	0	3
Ease of use of product	0	0	0	0	0	0	0	0
Proximity	1	0	0	0	0	0	0	1
Infrastructure	4	3	0	0	0	0	0	7
Remembering all required steps	4	0	0	0	0	0	0	4
Time	1	1	1	1	0	0	0	4

## Discussion

We identified a total of 78 studies that provided information on the possible determinants of clean birthing practices in LMIC – 60% of these were observational studies and 40% report evaluations of a specific behaviour change intervention. Of those studies where it was possible to identify determinants for individual clean practices, hand hygiene (*n* = 13) and clean cord care (n = 11) receive the greatest attention. Few of these studies focused on caregiver hand hygiene (n = 4) and birth surfaces (*n* = 5), despite both being potential mediators for the transmission of pathogens.

The vast majority of studies identified psychological capability (e.g. knowledge) and physical opportunity (e.g. access to infrastructure) as key determinants of clean birthing practices. This is surprising given the body of evidence suggesting that while knowledge and access to materials are necessary precursors for improved hygiene practices these determinants alone are not sufficient to ensure adoption of behaviours ([70–75]). Studies of handwashing behaviour outside of the perinatal period place a much greater emphasis on factors such as social opportunity and automatic motivation. Emotional drivers – such as nurture, disgust, and affiliation – have been associated with a range of hygiene behaviours in domestic contexts, and interventions targeting emotional drivers have been shown to have significant impact on behavioural outcomes [73, 76]. However, our review only identified one study that addressed motivational determinants of hand hygiene behaviour during the perinatal period [17].

Evidence from this review has identified significant gaps between both research on the determinants of clean birthing practices and efforts to improve those practices using modern approaches to behaviour change. Across intervention and observational studies, the majority of data on determinants of clean birthing practices is applied to a generalised set of behaviours (*n* = 50, 64%), rather than for specific practices (e.g. hand-hygiene, clean surface, clean cord cutting, tying or care) (*n* = 31, 40%). The implication is that what determines one clean birthing practice, such as handwashing with soap, is the same as what determines any other. However, studies in this review that do focus on specific behaviours suggest a greater distinction between what determines one clean behaviour and another. In particular, studies focusing on one or more specific behaviour place a larger emphasis on social opportunity when compared to studies that present clean birthing practices as a generalised set of behaviours. While efforts to combine clean birthing practices into a single suite of behaviours – such as the “six cleans” promoted by organizations such as WHO – may simplify policy and advocacy efforts, it also comes at the expense of understanding that each clean practice has a unique set of determinants. Interventions targeting multiple behaviours are often less effective than those targeting individual behaviours, or have a much smaller effect on behavioural outcomes [77].

This limited approach to behavioural determinants extends to the interventions that target clean birthing practices. The majority of the 31 studies reviewed that evaluate such interventions have not documented the target determinant for the intervention (*n* = 20). Of those that have done so (*n* = 11), only four have identified target determinants for one or more specific behaviour. For the reviewed intervention studies where target determinants are not stated, knowledge and adequate materials/supplies were implicit in the design. However, as indicated above, knowledge is often a poor predictor of behaviour when addressed in isolation of other opportunity and motivational determinants.

We recognize a number of limitations of this review. Due to resource constraints, the scope of the review was limited. Our search strategy limited the review to studies featuring in two databases and published since 2000 and, of those, studies written in English. The COM-B Framework, along with our methodology for ensuring inter-reviewer reliability, offered a simple and replicable framework for the classification of behavioural determinants. However, this classification process retains some inherent complexities. For example, several studies identify cost of materials as the determinant of several clean birthing practices. However, this can either be interpreted as an issue of absolute affordability (an issue of physical opportunity) or opportunity cost (an issue of reflective motivation). Furthermore, determinants are often interlinked. For example, this is seen in the possible interactions between insufficient knowledge and cultural beliefs. Our coding did not limit behaviours to a single determinant, allowing our analysis to capture these complex relationships. However, as a qualitative scoping review, our analysis identified any reported determinants that were associated with clean birthing practices; we are unable to assess which determinants have the greatest effect on behavioural outcomes nor are we able to quantify the strength of relationships. Given the current heterogeneity in how behavioural determinants are described in both observational and interventional studies, more precise or descriptive classifications of determinants may not be feasible.

## Conclusions

This review suggests that our current understanding of what drives or hinders the adoption of clean birthing practices in LMIC remains limited. Current literature on clean birthing practices focuses primarily on clean birthing practices as a generalised set of behaviours, often at the expense of understanding the drivers of the specific behaviours that enable safe, clean births. This lack of specificity is reflected across both observational and intervention studies. More in-depth understanding of specific behaviours, as well as targeted interventions to improve those behaviours, is needed. Current research has focused on a limited number of determinants. There remain critical data gaps around other possible determinants of clean birthing practices and how best these determinants can be leveraged to result in better, safer births. More theoretically informed formative research is required to further our understanding of these critical behaviours and grasp important behavioural motivators and social influences across different contexts. This work can support the design of more effective interventions to change these hygiene behaviours and improve neonatal and maternal health outcomes in LMIC.

## Supplementary information

**Additional file 1: Table S1.** Identified determinants mapped against COM-B model categories.

**Additional file 2: Table S2.** Frequency of mention of particular determinants among observational studies (*n* = 47).

**Additional file 3: Table S3.** Intervention studies where targeted determinant is not explicitly identified by the authors (*n* = 20).

**Additional file 4: Table S4.** Intervention studies where target determinants for clean birthing practices in general are identified (*n* = 7).

**Additional file 5: Table S5.** Intervention studies where target determinants for individual clean birthing practices are identified (*n* = 4).

## Data Availability

The datasets used and/or analysed during the current study are available from the corresponding author on reasonable request.

## Abbreviations

ANC: Antenatal Care
HCF: Healthcare facilities
LMIC: Low- and middle-income countries
TBA: Traditional birth attendant
UN: United Nations
WASH: Water, Sanitation and Hygiene
WHO: World Health Organisation
