# Supplementary material for: Determinants of clean birthing practices in low- and middle-income countries: a scoping review
Source: BMC Public Health. 2020 May 1;20:602. doi: 10.1186/s12889-020-8431-4 (PMC7195776; doi:10.1186/s12889-020-8431-4)
Supplement: Supplementary file 1 — Additional file 1: Table S1. Identified determinants mapped against COM-B model categories. [file 12889_2020_8431_MOESM1_ESM.docx]

**Additional Table 1: Identified determinants mapped against COM-B model categories**

| **Capability** | | **Motivation** | | **Opportunity** | |
| --- | --- | --- | --- | --- | --- |
| *Psychological* | *Physical* | *Reflective* | *Automatic* | *Social* | *Physical* |
| Confidence | Skills | Product acceptability | Automaticity | Social norms/collective behaviours | Adequate materials/ supplies |
| Education |  | Futility | Disgust | Social hierarchy/community influencers | Convenience |
| Guidelines |  | Fear of repercussions | Job motivation | Traditional/cultural beliefs | Ease of use |
| Knowledge |  | Practical considerations | Nurture | Trust in attendant | Infrastructure |
| Parity |  | Product cost | Sense of ownership |  | Remembering |
| Occupation |  | Willingness to pay | Sense of pride |  | Time |
|  |  |  | Teachable moments |  | Proximity |
|  |  |  |  |  |  |
