# Supplementary material for: Determinants of clean birthing practices in low- and middle-income countries: a scoping review
Source: BMC Public Health. 2020 May 1;20:602. doi: 10.1186/s12889-020-8431-4 (PMC7195776; doi:10.1186/s12889-020-8431-4)
Supplement: Supplementary file 2 — Additional file 2: Table S2. Frequency of mention of particular determinants among observational studies (n = 47). [file 12889_2020_8431_MOESM2_ESM.docx]

**Additional Table 2: Frequency of mention of particular determinants among observational studies (n=47)**

|  | **General**  **(n=23)** | **Hand hygiene** | | **Surface**  **(n=5)** | **Blade**  **(n=4)** | **Tie**  **(n=5)** | **Cord care**  **(n=10)** |
| --- | --- | --- | --- | --- | --- | --- | --- |
|  |  | ***Att.***  ***(n=7)*** | ***Caregiver***  ***(n=3)*** |  |  |  |  |
| **Capability** |  |  |  |  |  |  |  |
| **Psychological** |  |  |  |  |  |  |  |
| Educational level | 1  [1] |  |  |  |  |  | 1  [2] |
| Guidelines | 1  [3] | 1  [4] |  |  |  |  |  |
| Knowledge | 13  [3, 5-16] | 4  [17-20] | 3  [21-23] |  | 3  [13, 24, 25] | 4  [13, 20, 25, 26] | 4  [13, 27-29] |
| Occupation | 1  [5] |  |  |  |  |  |  |
| Parity | 1  [5] |  |  |  |  |  |  |
| **Physical** |  |  |  |  |  |  |  |
| Skills | 8  [1, 3, 6, 9-13] |  |  |  | 1  [25] | 1  [25] |  |
| **Motivation** |  |  |  |  |  |  |  |
| **Reflective** |  |  |  |  |  |  |  |
| Product acceptability |  |  |  |  |  |  | 1  [29] |
| Futility | 1  [30] |  |  |  |  |  |  |
| Practical considerations |  |  |  | 4  [20, 24, 31, 32] |  |  |  |
| Willingness to pay |  |  |  |  |  |  | 1  [33] |
| Product cost | 1  [34] |  |  |  |  |  |  |
| **Automative** |  |  |  |  |  |  |  |
| Automaticity |  |  | 1  [21] |  |  |  |  |
| Disgust |  |  | 1  [21] |  |  |  |  |
| Nurture |  |  | 1  [21] |  |  | 1  [35] | 4  [8, 27, 29, 35] |
| Ownership |  |  |  |  |  |  | 1  [29] |
| Sense of pride | 1  [36] |  |  |  |  |  |  |
| **Opportunity** |  |  |  |  |  |  |  |
| **Social** |  |  |  |  |  |  |  |
| Soc. hierarchies/ community influencers | 2  [15, 23] | 1  [37] | 1  [21] | 1  [20] |  |  | 4  [2, 27, 29, 35] |
| Traditional/cultural beliefs | 5  [12, 23, 36, 38, 39] |  |  | 4  [20, 31, 32, 40] | 2  [24, 41] | 1  [35] | 5  [8, 27, 29, 34, 42] |
| **Physical** |  |  |  |  |  |  |  |
| Adequate materials/supplies | 7  [3, 11, 14-16, 30, 43] | 5  [4, 18, 20, 37, 44] |  |  |  | 1  [20] |  |
| Convenience of the activity | 1  [15] | 2  [17, 18] |  |  |  |  |  |
| Infrastructure | 4  [11, 14, 45, 46] | 3  [4, 20, 44] |  |  |  |  |  |
| Time | 1  [30] | 1  [20] | 1  [23] | 1  [20] |  |  |  |

1. Shahjahan, M., et al., *Factors affecting newborn care practices in Bangladesh.* Paediatric and Perinatal Epidemiology, 2012. **26**(1): p. 13-18.

2. Abhulimhen-Iyoha, B.I. and M.O. Ibadin, *Determinants of cord care practices among mothers in Benin City, Edo State, Nigeria.* Nigerian Journal of Clinical Practice, 2012. **15**(2): p. 210-213.

3. Berhe, A.K., F. Tinsae, and G. Gebreegziabher, *Knowledge and practice of immediate newborn care among health care providers in eastern zone public health facilities, Tigray, Ethiopia, 2016.* Bmc Pediatrics, 2017. **17**.

4. Mehta, R., et al., *Infection control in delivery care units, Gujarat state, India: A needs assessment.* Bmc Pregnancy and Childbirth, 2011. **11**.

5. Berhe, M., et al., *Essential neonatal care utilization and associated factors among mothers in public health facilities of Aksum Town, North Ethiopia, 2016.* Plos One, 2017. **12**(4).

6. Crowe, S., et al., *Generating Insights from Trends in Newborn Care Practices from Prospective Population-Based Studies: Examples from India, Bangladesh and Nepal.* Plos One, 2015. **10**(7).

7. Devasenapathy, N., et al., *Association of antenatal care and place of delivery with newborn care practices: evidence from a cross-sectional survey in rural Uttar Pradesh, India.* Journal of Health Population and Nutrition, 2017. **36**.

8. Gurung, G., *Practices on immediate care of newborn in the communities of Kailali district.* Nepal Medical College Journal: NMCJ, 2008. **10**(1): p. 41-4.

9. Osrin, D., et al., *Cross sectional, community based study of care of newborn infants in Nepal.* BMJ, 2002. **325**(7372): p. 1063.

10. Acharya, D., et al., *Knowledge of Maternal and Newborn Care Among Primary Level Health Workers in Kapilvastu District of Nepal.* Annals of Medical and Health Sciences Research, 2016. **6**(1): p. 27-32.

11. Martinez, A.M., et al., *Barriers to neonatal care in developing countries: Parents' and providers' perceptions.* Journal of Paediatrics and Child Health, 2012. **48**(9): p. 852-858.

12. Masuno, K., et al., *Scaling up interventions to eliminate neonatal tetanus: Factors associated with the coverage of tetanus toxoid and clean deliveries among women in Vientiane, Lao PDR.* Vaccine, 2009. **27**(32): p. 4284-4288.

13. Rahi, M., et al., *Newborn care practices in an urban slum of Delhi.* Indian Journal of Medical Sciences, 2006. **60**(12): p. 506-13.

14. de Graft-Johnson, J., et al., *Cross-sectional observational assessment of quality of newborn care immediately after birth in health facilities across six sub-Saharan African countries.* BMJ open, 2017. **7**(3): p. e014680.

15. Beun, M.H. and S.K. Wood, *Acceptability and use of clean home delivery kits in Nepal: A qualitative study.* Journal of Health Population and Nutrition, 2003. **21**(4): p. 367-373.

16. Morrison, J., et al., *What Affects Clean Delivery Kit Utilization at Birth in Nepal? A Qualitative Study.* Asia-Pacific Journal of Public Health, 2015. **27**(2): p. NP1263-NP1272.

17. Asp, G., et al., *Challenges of immediate newborn care in maternity units in Lagos, Nigeria: An observational study.* Journal of Obstetrics and Gynaecology, 2011. **31**(7): p. 612-616.

18. Ji, G., H. Yin, and Y. Chen, *Prevalence of and risk factors for non-compliance with glove utilization and hand hygiene among obstetrics and gynaecology workers in rural China.* Journal of Hospital Infection, 2005. **59**(3): p. 235-241.

19. Ith, P., A. Dawson, and C. Homer, *Quality of maternity care practices of skilled birth attendants in Cambodia.* International Journal of Evidence-Based Healthcare, 2012. **10**(1): p. 60-7.

20. Shamba, D.D., et al., *Clean Home-delivery in Rural Southern Tanzania: Barriers, Influencers, and Facilitators.* Journal of Health Population and Nutrition, 2013. **31**(1): p. 110-117.

21. Greenland, K., et al., *The context and practice of handwashing among new mothers in Serang, Indonesia: a formative research study.* Bmc Public Health, 2013. **13**.

22. Opara, P., B. Alex-Hart, and T. Okari, *Hand-washing practices amongst mothers of under-5 children in Port Harcourt, Nigeria.* Paediatrics and International Child Health, 2017. **37**(1): p. 52-55.

23. Sinha, L.N., et al., *Newborn care practices and home-based postnatal newborn care programme - Mewat, Haryana, India, 2013.* Western Pacific Surveillance Response Journal, 2014. **5**(3): p. 22-9.

24. Khadduri, R., et al., *Household knowledge and practices of newborn and maternal health in Haripur district, Pakistan.* Journal of Perinatology, 2008. **28**(3): p. 182-187.

25. Baqui, A.H., et al., *Newborn care in rural Uttar Pradesh.* Indian Journal of Pediatrics, 2007. **74**(3): p. 241-247.

26. Moran, A.C., et al., *Newborn care practices among slum dwellers in Dhaka, Bangladesh: a quantitative and qualitative exploratory study.* Bmc Pregnancy and Childbirth, 2009. **9**.

27. Degefie, T., Y. Amare, and B. Mulligan, *Local understandings of care during delivery and postnatal period to inform home based package of newborn care interventions in rural Ethiopia: a qualitative study.* BMC International Health & Human Rights, 2014. **14**: p. 17.

28. Senarath, U., et al., *Factors associated with maternal knowledge of newborn care among hospital-delivered mothers in Sri Lanka.* Transactions of the Royal Society of Tropical Medicine and Hygiene, 2007. **101**(8): p. 823-830.

29. Walsh, S., et al., *Newborn cord care practices in Haiti.* Global Public Health, 2015. **10**(9): p. 1107-17.

30. Ith, P., et al., *Practices of skilled birth attendants during labour, birth and the immediate postpartum period in Cambodia.* Midwifery, 2013. **29**(4): p. 300-7.

31. Choudhury, N. and S.M. Ahmed, *Maternal care practices among the ultra poor households in rural Bangladesh: a qualitative exploratory study.* Bmc Pregnancy and Childbirth, 2011. **11**.

32. Thapa, N., et al., *High-risk childbirth practices in remote Nepal and their determinants.* Women & health, 2001. **31**(4): p. 83-97.

33. Coffey, P.S., et al., *Willingness to pay for a 4% chlorhexidine (7.1% chlorhexidine digluconate) product for umbilical cord care in rural Bangladesh: a contingency valuation study.* Bmc International Health and Human Rights, 2013. **13**.

34. Mrisho, M., et al., *Understanding home-based neonatal care practice in rural southern Tanzania.* Transactions of the Royal Society of Tropical Medicine and Hygiene, 2008. **102**(7): p. 669-678.

35. Amare, Y., *Umbilical cord care in Ethiopia and implications for behavioral change: a qualitative study.* BMC International Health & Human Rights, 2014. **14**: p. 12.

36. Ohaja, M. and J. Murphy-Lawless, *Unilateral collaboration: The practices and understandings of traditional birth attendants in southeastern Nigeria.* Women and Birth, 2017. **30**(4): p. E165-E171.

37. Moyer, C.A., et al., *Clean delivery practices in rural northern Ghana: a qualitative study of community and provider knowledge, attitudes, and beliefs.* Bmc Pregnancy and Childbirth, 2012. **12**.

38. Hopp, L.J., *Delivery practices, hygiene, birth attendance and neonatal infections in Karamoja, Uganda: a community-based study.* African Health Sciences, 2017. **17**(1): p. 7-13.

39. Sharkey, A., et al., *Maternal and newborn care practices in Sierra Leone: a mixed methods study of four underserved districts.* Health Policy and Planning, 2017. **32**(2): p. 151-162.

40. Thapa, N., et al., *Infant death rates and animal-shed delivery in remote rural areas of Nepal.* Social Science & Medicine, 2000. **51**(10): p. 1447-56.

41. Alam, M.A., et al., *Newborn umbilical cord and skin care in Sylhet District, Bangladesh: implications for the promotion of umbilical cord cleansing with topical chlorhexidine.* Journal of Perinatology, 2008. **28**: p. S61-S68.

42. Herlihy, J.M., et al., *Local Perceptions, Cultural Beliefs and Practices That Shape Umbilical Cord Care: A Qualitative Study in Southern Province, Zambia.* Plos One, 2013. **8**(11).

43. Lucey, O., M. Andriatsihosena, and M. Ellis, *Impact of a Training Package for Community Birth Attendants in Madagascar.* Journal of Tropical Pediatrics, 2011. **57**(1): p. 59-61.

44. Bazzano, A.N., et al., *Environmental Factors and WASH Practices in the Perinatal Period in Cambodia: Implications for Newborn Health.* International Journal of Environmental Research and Public Health, 2015. **12**(3): p. 2392-2410.

45. Benova, L., et al., *Where There Is No Toilet: Water and Sanitation Environments of Domestic and Facility Births in Tanzania.* Plos One, 2014. **9**(9).

46. Osrin, D., et al., *A Rapid Assessment Scorecard to Identify Informal Settlements at Higher Maternal and Child Health Risk in Mumbai.* Journal of Urban Health-Bulletin of the New York Academy of Medicine, 2011. **88**(5): p. 919-932.
