# Supplementary material for: Determinants of clean birthing practices in low- and middle-income countries: a scoping review
Source: BMC Public Health. 2020 May 1;20:602. doi: 10.1186/s12889-020-8431-4 (PMC7195776; doi:10.1186/s12889-020-8431-4)
Supplement: Supplementary file 3 — Additional file 3: Table S3. Intervention studies where targeted determinant is not explicitly identified by the authors (n = 20). [file 12889_2020_8431_MOESM3_ESM.docx]

**Additional Table 3: Intervention studies where targeted determinant is not explicitly identified by the authors (n=20)**

| **Authors**  **Location** | **Intervention** | | **Target determinants implied by expected outputs (key below)** | | | | | | | | | | | | | |
| --- | --- | --- | --- | --- | --- | --- | --- | --- | --- | --- | --- | --- | --- | --- | --- | --- |
|  | **Description** | **Expected outputs (clean delivery practices)** |  | |  |  |  | | |  | | | |  | | |
|  |  |  | **Confidence (=1)** | **Knowledge (n=19)**  **(** | **Skills (n=7)** | **Impunity (n=3)** | **Job motivation (n=2)** | **Ownership (=n3_** | **Teachable moment moment (n=1)** | **Social hierarchy/community influencers (n=4)** | **Trust in attendant (n=1_** | **Traditional/cultural beliefs (n=0)** | **Collective behaviours/social norms (n=0)** | **Adequate materials/supplies (n=8)** | **Proximity (n=1)** | **Remembering all required steps (n=3)** |
| [1]  *Egypt* | **To improve use of clean delivery kits.** Primary health-centre-based. Clean delivery kit (CDK) distribution to traditional birth attendants (TBAs) from primary care facilities; Training of mothers and birth attendants on kit importance and use | - Maternal ownership and use of CDK - Knowledge of importance of CDK and how to use it by mothers and birth attendants |  | ✓ | ✓ |  |  |  |  |  |  |  |  | ✓ |  |  |
| [2]  *Rural Ethiopia* | **To improve use of antenatal and delivery services.** Community-based. Creation of Health Posts (HPs) within each Kabele with trained local Health Extension Workers (HEWs) known to the community, who provide ANC services, advise mothers on birth preparedness and pregnancy/labour-related warning signs/symptoms, promote institutional delivery, manage normal deliveries and identify/refer high risk pregnancies. | - Close/convenient access to clean and safe delivery services at HPs - Maternal knowledge of birth preparedness - Maternal trust in attendant - HEW and TBA knowledge and skills to manage normal deliveries |  | ✓ | ✓ |  |  |  |  |  | ✓ |  |  |  | ✓ |  |
| [3]  *Rural Pakistan* | **To reduce perinatal and neonatal mortality.** Community-based. Three components: Lady health worker (LHW) enhanced training, TBA basic newborn care training, creation of volunteer-based community health committees (CHCs) in villages in liaison with LHWs. LHW trained to: Promote ANC, facility births and careseeking; educate mothers on use of CDKs, immediate newborn care and identifying danger signs. CDKs provided. No specifics provided on TBA training. CHCs created to: promote maternal and newborn care in village, advocate to community elders/local political leaders, organise emergency transport using local resources. | - Maternal knowledge of birth preparedness and importance of ANC - LHW ownership and use of CDK - Institutional delivery - Community influencers knowledge and local promotion of safe maternal and newborn care |  | ✓ |  |  |  |  |  | ✓ |  |  |  | ✓ |  |  |
| [4]  *Egypt* | **To reduce morbidity due to newborn umbilical cord and maternal puerperal infections.** Primary health-centre-based. CDK distribution by primary-care facilities and birth attendant training on kit use. | - Birth attendant ownership of CDK - Birth attendant knowledge of importance of CDK and how to use it |  | ✓ |  |  |  |  |  |  |  |  |  | ✓ |  |  |
| [5]  *Kenya* | **To increase use of maternal health services and improve household hygiene and nutrition through distribution of water treatment products, soap, protein-fortified flour, and clean delivery kits.** Primary and secondary health-centred-based. Intervention components: 1-week training of nurses and clinical officers to improve health facility quality of care (managing obstetric emergencies, neonatal resuscitation, patient-centred care, rapid syphilis testing and treatment, water treatment, and handwashing with soap); provision of bulb syringe for neonatal suctioning, ambu-bag for neonatal resuscitation, and handwashing and drinking water stations at each facility; several items offered to mothers at ANC visits to incentivise attendance at maternal health services (free hygiene kits, water treatment solution and soap) at 1^st^ and 3^rd^ visits, protein-fortified flour at 2^nd^ and 3^rd^ ANC visits, clean delivery kit at time of health-facility delivery, free syphilis screening and treatment at 1^st^ ANC visit); reproductive health education for women by local providers. | - Increased maternal use of maternal health services - Increased maternal access to soap through programme distribution, leading to increased maternal handwashing with soap - Increased maternal knowledge of the importance of handwashing with soap for newborn health, leading to increased maternal handwashing with soap |  | ✓ |  |  |  |  |  |  |  |  |  | ✓ |  |  |
| [6]  *Brazil* | **To improve families’ knowledge of newborn care.** Primary health-centre-based. Educational package for mother-infant-family host group. Activities performed by a nurse once/week, 40 minutes (six meetings total). Dialogical educational model, giving family opportunity to exchange experiences and clarify doubts. Topics: oral and personal hygiene, cord care, cramping, sun bathing, breastfeeding, children’s health booklet, physiological eliminations | - Caregiver knowledge of personal hygiene and cord care - Caregiver confidence in carrying out clean cord care | ✓ | ✓ |  |  |  |  |  |  |  |  |  |  |  |  |
| [7]  *Bangladesh* | **To reduce maternal postpartum infection.** Community-based. **T**BA training in the 'three cleans' (handwashing with soap (HWWS), clean cord care, clean surface) | TBA knowledge of good practice and handwashing technique |  | ✓ | ✓ |  |  |  |  |  |  |  |  |  |  |  |
| [8]  *India* | **To improve quality of childbirth services in high caseload public health facilities.** Primary/secondary-health-facility-based. 1. Health staff training (senior doctors and district managers on role of evidence-based care, barriers, planning for improved quality; senior nurse-midwives/nurse-midwives on skilled birth attendance); 2. Regular facility assessment, feedback, training, action cycle (score the facility, feed shortcomings to clinical and managerial staff, discuss ways to overcome them, share report cards with block and district health officials, discuss in district/state review meetings, spot orientation–training, address gaps in equipment and supplies. | - Health care staff knowledge of evidence -based care practices for childbirth - Health care staff motivation (through participatory approaches and continual monitoring and feedback) to adhere to these practices. |  | ✓ | ✓ | ✓ |  | ✓ |  |  |  |  |  | ✓ |  |  |
| [9]  *Pakistan* | **To reduce maternal and neonatal mortality.** Primary-health-centre and community-based. CDK distribution to TBAs from primary care facilities, TBA training, LHW training to support TBAs and record data**.** 3-day training using picture cards with advice on antepartum, intrapartum and postpartum care: how to conduct clean delivery, use of CDK, when to refer women for emergency care and care of the newborn. TBAs to visit women ≥3 times during pregnancy to check for dangerous signs, and promote emergency careseeking where needed. | - Birth attendant ownership of CDK - Birth attendant knowledge of how to conduct clean delivery. - Improved standing of TBAs among their clients due to provision of CDKs - Increased links between TBAs and primary care facilities |  | ✓ |  |  |  |  |  | ✓ |  |  |  | ✓ |  |  |
| [10]  *Nepal* | **To improve knowledge of birth preparedness and essential newborn care (ENC).** Community-based. Training of female community health volunteer to provide antenatal care (ANC) counselling or health education to new mothers, and identify, assess and provide basic management of sick newborn. | Maternal knowledge of birth preparedness and ENC |  | ✓ |  |  |  |  |  |  |  |  |  |  |  |  |
| [11]  *Ghana* | **To improve newborn survival via home-visits.** Community-based. Training community-based surveillance volunteers to undertake two home visits of women, during pregnancy and after birth, to promote ENC, identify and treat/refer danger signs in newborns, counsel family on these, promote careseeking. Community-wide meetings chaired by community chiefs to: introduce importance of newborn care, explain intervention, discuss importance of community support, present trained CBSVs with branded materials. Refresher training two months later. | - Maternal knowledge of ENC - Community influencers knowledge and local promotion of ENC - Community sense of ownership of intervention |  | ✓ |  |  |  | ✓ |  | ✓ |  |  |  |  |  |  |
| [12]  *India* | **To improve maternal knowledge of birth preparedness and ENC and ensure provision of adequate materials.** Community-based. Water treatment and hygiene promotion provided during ANC visits. Safe water storage containers, WaterGuard water treatment solution and soap provided, and hygiene education was delivered. | - Maternal knowledge of birth preparedness and ENC - Households have adequate materials and supplies to wash hands |  | ✓ |  |  |  |  |  |  |  |  |  | ✓ |  |  |
| [13] *India* | **To improve supply of and demand for services delivered during 1,000-day window through health system training and community outreach.** Facility and community-based. Ananya intervention had two components. First, to strengthen supply and delivery of essential family health and infectious disease interventions through training, mobilizing, and monitoring government Community Health Workers (CHWs) and empowering them with job-aids and tools to increase quantity, quality, and ultimately, effectiveness of home visits for RMNH screenings and services to increase demand for services. Tools included the mobile kunji, an interactive voice response-based mobile service and a printed deck of cards covering messages related to 10 life-saving RNMCH behaviours to help enhance CHWs’ counselling of families, and a mobile training course for CHWs to expand and refresh their knowledge of life-saving RMNCH behaviours.  Second, to improve demand for services through changing behaviours, social norms, and self-efficacy to support family health. | - CHW knowledge of birth preparedness and ENC - Improved outreach by CHWs - Maternal knowledge of birth preparedness and essential newborn care (ENC) - Skilled attendance at birth - Changes in community social norms towards enforcing birth preparedness and ENC. |  | ✓ | ✓ |  |  |  |  |  |  |  |  |  |  |  |
| [14]  *29 countries (6 regions)* | **To improve skilled birth attendant adherence to safe childbirth practices, from admission to discharge.** Facility-based. WHO Safe Childbirth Checklist implementation through training, coaching and supervision of end-users. Implementation of checklist by 34 institutions in 200 sites, 29 countries. Unclear how checklist was introduced, what activities formed the intervention. | - Improvements in quality of care, to complement provision of life-saving interventions to reduce maternal mortality. - Birth attendants remember each step they are required to carry out. |  |  |  |  |  |  |  |  |  |  |  |  |  | ✓ |
| [15]  *Malawi, Nepal, Bangladesh,Uganda.* | **To improve maternal newborn care practices.** Community-based. CHW trained on maternal and newborn care and instructed to make 2-4 home visits during pregnancy and 2-4 home visits after the birth. | - Maternal knowledge of newborn care practices. |  | ✓ |  |  |  |  |  |  |  |  |  |  |  |  |
| [16]  *India* | **To improve childbirth practices.** Facility-based. WHO Safe Childbirth Checklist implementation through training of end-users, and coaching and supervision. (1) Engagement of local administrative and clinical leaders and identification of facility-based implementation leads; (2) Education about childbirth safety principles, deficiencies in current practice, and how to use the Checklist; (3) Execution beginning with one week of simulation and supervised practice and (4) Evaluation and ongoing monitoring. Hospital-based implementation leads selected by facility and trained by investigators. They introduced the checklist programme to staff during the one-day learning session and monitored its ongoing use. Learning was supported by written materials, lectures, an instructional video and hands-on simulation. | - Local administrative and clinical leaders become central to change - Birth attendants have knowledge about childbirth safety principles and how to use the Safe Childbirth Checklist - Birth attendants remember each step they are required to carry out, from admission to discharge. - Birth attendants are motivated to use the checklist through coaching and supervision |  | ✓ |  | ✓ |  |  |  | ✓ |  |  |  |  |  | ✓ |
| [17] *Uganda* | **To improve the use of recommended intrapartum and postnatal interventions.** Secondary-health-facility-based. Six activities: Dissemination workshops, reinforced through reminder development, birth simulation sessions, team building, case reviews, and academic visits to wards. Booklet explaining the nine practices, checklist, and monitoring cards for other activities produced for dissemination. Group of health providers, identified as opinion leaders selected and trained in 2-day workshop on how to implement the activities. | - SBA knowledge and skills about recommended intrapartum and postnatal interventions - SBAs remember each step they are required to carry out - SBA increased job motivation through team building and participatory approaches |  | ✓ | ✓ |  | ✓ |  |  |  |  |  |  |  |  | ✓ |
| [18]  *India, Bangladesh, Nepal, Malawi* | **To improve key antenatal, delivery and postnatal behaviours.** Community-based. Intervention consisted of participatory learning and action (PLA) approaches via women's groups. One ambition was to reach pregnant women through these groups, building on a previous meta-analysis that assessed the effect of such groups on neonatal mortality and found the effect of the intervention was partly dependent on the proportion of pregnant women attending groups, and on the population coverage of the groups. | - Maternal knowledge of antenatal, delivery and postnatal behaviours - Maternal sense of ownership of solutions (through participatory approaches) |  | ✓ |  |  |  | ✓ | ✓ |  |  |  |  |  |  |  |
| [19]  *Uganda, Tanzania* | **To improve coverage and quality of essential maternal and newborn services.** Community and facility-based. Systematic and collaborative quality improvement approach targeting district managers, health facility staff and community health workers on coverage and quality of essential maternal and newborn health services. Every quarter, learning sessions occurred to remind participants about QI techniques, present new topics for improvement, review progress and share learnings across teams. Between learning sessions, during implementation, regular mentoring of QI teams by project staff and district managers. Learning sessions supported with periodic feedback of continuous household and health facility surveys results. | - District health managers, health facility staff and community health workers knowledge and skills to implement quality essential maternal and newborn health services - Staff job motivation through continual mentoring, and continual monitoring and feedback of progress |  | ✓ | ✓ | ✓ | ✓ |  |  |  |  |  |  |  |  |  |
| [20]  *Tanzania* | **To prevent cord infection and puerperal sepsis.** Community and facility-based. Intervention incorporated education of mothers about the "six cleans" and how to use a clean delivery kit, with distribution of a CDK by village health workers. | - Maternal knowledge of the ‘six cleans’ and correct use of CDK - Maternal ownership of a CDK |  | ✓ |  |  |  |  |  |  |  |  |  | ✓ |  |  |

1. Balsara, Z.P., et al., *Impact of clean delivery kit use on clean delivery practices in Beni Suef Governorate, Egypt.* Journal of Perinatology, 2009. **29**(10): p. 673-679.

2. Bayou, N.B. and Y.H. Gacho, *Utilization of clean and safe delivery service package of health services extension program and associated factors in rural kebeles of Kafa Zone, Southwest Ethiopia.* Ethiopian Journal of Health Sciences, 2013. **23**(2): p. 79-89.

3. Bhutta, Z.A., et al., *Improvement of perinatal and newborn care in rural Pakistan through community-based strategies: a cluster-randomised effectiveness trial.* Lancet, 2011. **377**(9763): p. 403-412.

4. Darmstadt, G.L., et al., *Impact of Clean Delivery-kit use on Newborn Umbilical Cord and Maternal Puerperal Infections in Egypt.* Journal of Health Population and Nutrition, 2009. **27**(6): p. 746-754.

5. Fagerli, K., et al., *Impact of the Integration of Water Treatment, Hygiene, Nutrition, and Clean Delivery Interventions on Maternal Health Service Use.* The American journal of tropical medicine and hygiene, 2017. **96**(5): p. 1253-1260.

6. Gomes, A.L.M., et al., *Family knowledge on newborn care.* Revista Da Rede De Enfermagem Do Nordeste, 2015. **16**(2): p. 258-265.

7. Goodburn, E.A., et al., *Training traditional birth attendants in clean delivery does not prevent postpartum infection.* Health Policy & Planning, 2000. **15**(4): p. 394-9.

8. Iyengar, K., et al., *Adherence to evidence based care practices for childbirth before and after a quality improvement intervention in health facilities of Rajasthan, India.* Bmc Pregnancy and Childbirth, 2014. **14**.

9. Jokhio, A.H., H.R. Winter, and K.K. Cheng, *An intervention involving traditional birth attendants and perinatal and maternal mortality in Pakistan.* New England Journal of Medicine, 2005. **352**(20): p. 2091-2099.

10. Khanal, S., W.D. Zhang, and S. Khanal, *The Efficacy of Community Based Intervention in Newborn Care Practices and Neonatal Illness Management in Morang District of Nepal.* Life Science Journal-Acta Zhengzhou University Overseas Edition, 2009. **6**(4): p. 34-40.

11. Kirkwood, B.R., et al., *Effect of the Newhints home-visits intervention on neonatal mortality rate and care practices in Ghana: a cluster randomised controlled trial.* Lancet, 2013. **381**(9884): p. 2184-92.

12. Loharikar, A., et al., *Long-term Impact of Integration of Household Water Treatment and Hygiene Promotion with Antenatal Services on Maternal Water Treatment and Hygiene Practices in Malawi.* American Journal of Tropical Medicine and Hygiene, 2013. **88**(2): p. 267-274.

13. McDougal, L., et al., *Making the continuum of care work for mothers and infants: Does gender equity matter? Findings from a quasi-experimental study in Bihar, India.* PLoS ONE [Electronic Resource], 2017. **12**(2): p. e0171002.

14. Perry, W.R.G., et al., *Implementing the WHO Safe Childbirth Checklist: lessons from a global collaboration.* Bmj Global Health, 2017. **2**(3).

15. Sitrin, D., et al., *Improving newborn care practices through home visits: lessons from Malawi, Nepal, Bangladesh, and Uganda.* Glob Health Action, 2015. **8**: p. 23963.

16. Spector, J.M., et al., *Improving Quality of Care for Maternal and Newborn Health: Prospective Pilot Study of the WHO Safe Childbirth Checklist Program.* Plos One, 2012. **7**(5).

17. Spira, C., et al., *Improving the quality of maternity services in Uganda through accelerated implementation of essential interventions by healthcare professional associations.* International Journal of Gynecology & Obstetrics, 2017. **139**(1): p. 107-113.

18. Seward, N., et al., *Effects of women's groups practising participatory learning and action on preventive and care-seeking behaviours to reduce neonatal mortality: A meta-analysis of cluster-randomised trials.* PLoS Medicine / Public Library of Science, 2017. **14**(12): p. e1002467.

19. Waiswa, P., et al., *Effects of the EQUIP quasi-experimental study testing a collaborative quality improvement approach for maternal and newborn health care in Tanzania and Uganda.* Implementation Science, 2017. **12**.

20. Winani, S., et al., *Use of a clean delivery kit and factors associated with cord infection and puerperal sepsis in Mwanza, Tanzania.* Journal of Midwifery & Womens Health, 2007. **52**(1): p. 37-43.
