# Supplementary material for: Determinants of clean birthing practices in low- and middle-income countries: a scoping review
Source: BMC Public Health. 2020 May 1;20:602. doi: 10.1186/s12889-020-8431-4 (PMC7195776; doi:10.1186/s12889-020-8431-4)
Supplement: Supplementary file 4 — Additional file 4: Table S4. Intervention studies where target determinants for clean birthing practices in general are identified (n = 7). [file 12889_2020_8431_MOESM4_ESM.docx]

**Additional Table 4: Intervention studies where target determinants for clean birthing practices in general are identified (n=7)**

| **Authors**  **Location** | **Intervention description** | **Target determinants (see key)** | | | | | | | | | | | | | | |
| --- | --- | --- | --- | --- | --- | --- | --- | --- | --- | --- | --- | --- | --- | --- | --- | --- |
|  |  | **Description** |  | |  |  |  | | |  | | | |  | | |
|  |  |  | **Confidence (n=0)** | **Knowledge (n=4)** | **Skills (n=1)** | **Impunity (n=1)** | **Job motivation (n=1)** | **Ownership (n=2)** | **Teachable moment moment (n=1)** | **Community influencers (n=1)** | **Trust in attendant (n=0)** | **Traditional/cultural beliefs (n=0)** | **Collective behaviours/social norms (n=0)** | **Adequate materials/supplies (n=0)** | **Proximity (n=0)** | **Remembering all required steps (n=1)** |
| [1]  *Uganda* | **To improve use of maternal and newborn services and care practices through participatory multisectoral maternal and newborn intervention.** Community and facility-based. The MANIFEST intervention: community mobilization and empowerment component to stimulate demand for services (home visits by CHWs, radio-based health education, promotion of saving through saving groups, promotion of partnerships with local transporters), and health provider and management capacity-building component to strengthen delivery of quality maternal and newborn health services (emergency obstetric and newborn care refresher training, mentorship and support supervision of primary health workers, certificate course in health services management for health managers, recognition of best performing facilities and managers. | Knowledge: Community-based strategies involving CHW and TBA training have resulted in increased awareness of maternal and newborn health and danger signs, increased use of ANC and facility delivery, and increased newborn care practices.  Community influencers: Community mobilisation and use of community support groups have also resulted in increased awareness and knowledge about maternal and newborn health and facility deliveries |  | ✓ |  |  |  |  |  |  |  |  |  |  |  |  |
| [2]  *Benin* | **To examine the effect of a job-aids-focused intervention on quality of counselling and maternal understanding of care for mothers and newborns**. Facility-based. Intervention providers trained to use job-aids and provided implementation support to enhance antenatal care messaging effectiveness | Knowledge: Intervention geared towards improving quality of counselling communication through use of visuals. Communication provided antenatally is an effective strategy to improve maternal understanding and health practices. Yet poor quality of communication may be leading to low maternal knowledge. |  | ✓ |  |  |  |  |  |  |  |  |  |  |  |  |
| [3]  *Nepal* | **To assess impact of community-based participatory intervention on perinatal care practices, emergency care-seeking and neonatal mortality rates.** Community-based. Intervention: female facilitator convened monthly women's group meetings and supported groups through an action-learning cycle in which they identified local perinatal problems and formulated strategies to address them. Facilitator rather than teacher, with abilities and training in participatory community techniques. None of the facilitators had a health background but had brief training in perinatal health issues. | Participatory approach to intervention design: in contrast to models based on direct education by health workers, which have shown no effect on infant care practices and care-seeking behaviour after delivery. |  |  |  |  |  | ✓ |  |  |  |  |  |  |  |  |
| [4]  *Tanzania* | **To improve newborn care practices through a home-based counselling intervention by community volunteers.** Community-based. Mtunze Mtoto Mchanga (protect your newborn baby) intervention: 3 home visits to women and families during pregnancy and 2 visits in first few days of infant's life, to promote key behaviours. Key counselling msgs selected on basis of frequency of behaviour, feasibility of change, likely impact on survival on the basis of evidence published at the time. | Knowledge: Maternal knowledge of newborn care practices.  Life-stage: Pregnancy and immediately after as teachable moments. |  | ✓ |  |  |  |  | ✓ |  |  |  |  |  |  |  |
| [5]  *India* | **To improve neonatal survival through a women’s group intervention involving participatory learning and action.** Community-based. Local female facilitators guide women’s groups through a cycle of activities involving participatory learning and action, during which women identify, prioritize and analyse local maternal and neonatal health problems and subsequently devise and implement strategies to address them. | Participatory approaches: The success and sustainability of community-based programmes for improving maternal and neonatal health require the active involvement of women, families and community health-care workers, yet the strategies used to engage these groups are often externally driven and top-down. Community mobilization, defined here as a process through which communities plan and act together to address health problems, is an essential component of programmes for improving maternal and child health. |  |  |  |  |  | ✓ |  |  |  |  |  |  |  |  |
| [6]  *India* | **To improve skilled birth attendant adherence to safe childbirth practices, from admission to discharge.** Primary-health-centre-based. BetterBirth, 8-month coaching-based implementation of the Safe Childbirth Checklist. Coaches, unaffiliated with the facilities and trained to apply a standard behaviour-change framework during site visits to motivate birth attendants to use the checklist, and identify, understand, and resolve barriers to quality care. ≥1 staff member/facility served as quality coordinator and local champion for checklist use and provided continued coaching. | Remembering all required steps: essential birth practices are well documented but often not performed. Intervention addresses the “know-do” gap through a checklist job-aid that supports routine adherence to evidence-based practices.  Fear of repercussions/job motivation: Job aids alone have not been found to improve health care workers’ performance. Peer coaching based on feedback about adherence to checklist was used as additional strategy to promote behaviour change (BC).  Ensuring buy-in from the larger health care system: |  |  |  | ✓ | ✓ |  |  | ✓ |  |  |  |  |  | ✓ |
| [7]  *Sri Lanka* | **To improve practice of ENC in obstetric units through training program for care providers.** Secondary-health-facility-based. Intervention: 4-day training program on ENC for doctors, nurses, and midwives of the obstetric units, to increase knowledge of ENC and develop the corresponding skills among midwives, nurses, and doctors in obstetric units. Findings of pre-intervention study led to formulation of learning objectives. 15-module training manual compiled by the investigators in consultation with an expert group, mainly based on WHO Training Modules. Adapted teaching aids were also employed. | Knowledge and skills: There is sufficient evidence regarding the impact of health worker training on favourable neonatal health outcomes in community settings. |  | ✓ | ✓ |  |  |  |  |  |  |  |  |  |  |  |

1. Ekirapa-Kiracho, E., et al., *Effect of a participatory multisectoral maternal and newborn intervention on maternal health service utilization and newborn care practices: a quasi-experimental study in three rural Ugandan districts.* Global Health Action, 2017. **10**.

2. Jennings, L., et al., *Antenatal counseling in maternal and newborn care: use of job aids to improve health worker performance and maternal understanding in Benin.* BMC Pregnancy & Childbirth, 2010. **10**: p. 75.

3. Manandhar, D.S., et al., *Effect of a participatory intervention with women' groups on birth outcomes in Nepal: cluster-randomised controlled trial.* Lancet, 2004. **364**(9438): p. 970-979.

4. Penfold, S., et al., *Effect of home-based counselling on newborn care practices in southern Tanzania one year after implementation: a cluster-randomised controlled trial.* Bmc Pediatrics, 2014. **14**.

5. Roy, S.S., et al., *Improved neonatal survival after participatory learning and action with women's groups: a prospective study in rural eastern India.* Bulletin of the World Health Organization, 2013. **91**(6): p. 426-433.

6. Semrau, K.E., et al., *Outcomes of a Coaching-Based WHO Safe Childbirth Checklist Program in India.* New England Journal of Medicine, 2017. **377**(24): p. 2313-2324.

7. Senarath, U., D.N. Fernando, and I. Rodrigo, *Effect of training for care providers on practice of essential newborn care in hospitals in Sri Lanka.* Jognn-Journal of Obstetric Gynecologic and Neonatal Nursing, 2007. **36**(6): p. 531-541.
