# Supplementary material for: Determinants of clean birthing practices in low- and middle-income countries: a scoping review
Source: BMC Public Health. 2020 May 1;20:602. doi: 10.1186/s12889-020-8431-4 (PMC7195776; doi:10.1186/s12889-020-8431-4)
Supplement: Supplementary file 5 — Additional file 5: Table S5. Intervention studies where target determinants for individual clean birthing practices are identified (n = 4). [file 12889_2020_8431_MOESM5_ESM.docx]

**Additional Table 5: Intervention studies where target determinants for individual clean birthing practices are identified (n=4)**

| **Authors**  **Location** | **Intervention description** | **Target behaviour** | **Target determinants (see key)** | | | | | | | | | | | | | | |
| --- | --- | --- | --- | --- | --- | --- | --- | --- | --- | --- | --- | --- | --- | --- | --- | --- | --- |
|  |  |  | **Description** |  | |  |  |  | | |  | | | |  | | |
|  |  |  |  | **Confidence (n=0)** | **Knowledge (n=2)** | **Skills (n=1)** | **Impunity (n=0)** | **Job motivation (n=0)** | **Ownership (n=1)** | **Teachable moment moment (n=1)** | **Community influencers (n=2)** | **Trust in attendant (n=0)** | **Traditional/cultural beliefs (n=2)** | **Collective behaviours/social norms** | **Adequate materials/supplies (n-=1)** | **Proximity (n=0)** | **Remembering all required steps (n=0)** |
| [1]  *Bangladesh* | **To determine whether exposure to an intensive handwashing intervention in the perinatal period was associated with improved maternal handwashing behaviour vs. perinatal period.** Community-based. Intervention: Interactive educational programme to promote handwashing with soap at critical events expected to interrupt pathogen transmission to the neonate. Handwashing encouraged as a way for mother to nurture child by providing a safe and healthy environment. Hardware for three different handwashing stations also provided as well as HH poster that pictorially describes handwashing events. | Caregiver hand hygiene | Life-stage: Pregnancy as a teachable moment. A period of increased emotion, change in social roles, increased perception of health risk.  Participatory approach: Behaviour change (BC) communicators trained on motivational interviewing to encourage participant and family to actively engage in conversation about benefits and motivators, identify barriers and find acceptable solutions. Participants and families encouraged to determine best placement for handwashing materials in home. |  |  |  |  |  | ✓ | ✓ |  |  |  |  |  |  |  |
| [2]  *India* | **To improve newborn care practices and reduce neonatal mortality through a community-based intervention.** Community-based. Intervention group 1: Essential newborn care (ENC) package. Group 2: ENC plus use of liquid crystal hypothermia indicator (ThermoSpot). Community-based intervention delivered by community health workers via collective meetings and two antenatal and two postnatal household visitations. Multi-level strategy developed to target community stakeholders, newborn stakeholders, and households (HH) with immediate support groups. At each level, target group consisted of individuals with key roles as influencers, decision makers, supporters, practitioners of newborn care and normative behaviour within community. BC approach based on trust and developed as participatory process of community engagement to lead to BC “through path of least social, cultural, economic and spiritual resistance to change". Messages designed to promote improved practices that aligned with cultural values and traditions. Intervention design identified high-risk behaviours, individuals with key roles in the practice and continuation of these behaviours, and potential barriers, opportunities and factors affecting behaviour change. | Attendant hand hygiene | Social hierarchies/community influencers: Individual behaviours influenced by collective behaviours and social norms, and sustained by complex multilevel network of relationships within community. Mother found to be primary care provider but usually not empowered to make decisions. Mother-in-law usually key decision-maker on newborn-care practices. Male HH members, incl. father in law and husband, controlled access to HH, made financial and logistical arrangements and influenced care-seeking decisions.  Traditional/cultural beliefs: The delivery process and the newborn are considered unclean and “polluting”, so birth attendants usually do not wash their hands.  Collective behaviours/social norms |  |  |  |  |  |  |  | ✓ |  | ✓ | ✓ |  |  |  |
|  |  | Clean blade | Social hierarchies/community influencers: Individual behaviours influenced by collective behaviours and social norms, and sustained by complex multilevel network of relationships within community. Mother found to be primary care provider but usually not empowered to make decisions. Mother-in-law usually key decision-maker on newborn-care practices. Male HH members, incl. father in law and husband, controlled access to HH, made financial and logistical arrangements and influenced care-seeking decisions.  Knowledge and skills: Domins are not trained in hygienic cord cutting. |  | ✓ | ✓ |  |  |  |  | ✓ |  |  |  |  |  |  |
| [3]  *Nicaragua* | **To reduce neonatal sepsis through improving correct use of disinfectants; appropriate hand hygiene and identification of cases of and risk factors for neonatal sepsis.** Facility-based. Intervention: develop national guidelines on correct use of disinfectants and hand hygiene; train medical staff on these; revise basic medical supply list to support appropriate antisepsis; define package of diagnostic tests for neonatal sepsis; systematically measure compliance. | Attendant hand hygiene | Knowledge: Many staff were unfamiliar with the concepts of hand washing and alcohol gel as an acceptable antiseptic.  Adequate materials/supplies: Alcohol gel not included on MINSA’s basic supplies list and therefore not available at many facilities. |  | ✓ |  |  |  |  |  |  |  |  |  | ✓ |  |  |
| [4]  *Kenya, Tanzania* | **To evaluate the effect of a simple health promotion programme, designed in consultation with local community and carried out by local women, in reducing neonatal tetanus among a Maasai community.** Community-based. Health promotion intervention: Peer group education, prenatal monitoring, delivery, postpartum follow-up, referral where necessary, CDK distribution to TBAs. Delivered by locally recruited TBAs. Training emphasised use of clean water vs cow dung on the freshly cut cord. | Clean cord care | Traditional/cultural beliefs: "Culturally integrated" approach to design and delivery. Substitutes to cow-dung proposed by community spiritual leaders - cow dung symbolic of Maasai way of life. Water significant because Maasai believe that cows originally came from water. |  |  |  |  |  |  |  |  |  | ✓ |  |  |  |  |

1. Kamm, K.B., et al., *Is pregnancy a teachable moment to promote handwashing with soap among primiparous women in rural Bangladesh? Follow-up of a randomised controlled trial.* Tropical Medicine & International Health, 2016. **21**(12): p. 1562-1571.

2. Kumar, V., et al., *Effect of community-based behaviour change management on neonatal mortality in Shivgarh, Uttar Pradesh, India: a cluster-randomised controlled trial.* Lancet, 2008. **372**(9644): p. 1151-1162.

3. Lopez, S., et al., *Quality in practice: preventing and managing neonatal sepsis in Nicaragua.* International Journal for Quality in Health Care, 2013. **25**(5): p. 599-605.

4. Meegan, M.E., et al., *Effect on neonatal tetanus mortality after a culturally-based health promotion programme.* The Lancet, 2001. **358**(9282): p. 640-641.
